# Enhancing Neurodevelopmental Outcomes in Preterm Infants Through the Sensory Development Care Map

**DOI:** 10.3390/children12020192

**Published:** 2025-02-06

**Authors:** Lisa Sampson, Maureen Luther, Asaph Rolnitsky, Eugene Ng

**Affiliations:** 1 Dan Women and Babies Program, Sunnybrook Health Sciences Centre, Toronto, ON M4N 3M5, Canada; lisa.sampson@sunnybrook.ca (L.S.); maureen.luther@sunnybrook.ca (M.L.); asaph.rolnitsky@sunnybrook.ca (A.R.); 2 Department of Paediatrics, University of Toronto, Toronto, ON M5G 1X8, Canada

**Keywords:** preterm birth, NICU, sensory development, parents, neurodevelopmental outcomes

## Abstract

**Background/Objectives:** Preterm infants are at a high risk of neurodevelopmental impairments due to immature brain development and the stressors of the neonatal intensive care unit (NICU) environment. To improve outcomes, incorporating a neuropromotion strategy by promoting nurturing encounters (NEs) is essential. **Methods:** In this 48-bed tertiary perinatal care center, an informal survey showed that staff lacked consistent knowledge about sensory neurodevelopment, while parents expressed a need for clearer guidance. This paper describes the development and implementation of the Sensory Developmental Care Map (SDCM) as part of a larger quality-improvement initiative. The SDCM is an educational tool designed to guide NICU staff and families in providing neuroprotective and neuropromotive care, based on the infant’s gestational age (GA). The SDCM was created by integrating evidence on sensory development across GAs and providing practical strategies to promote positive sensory input while protecting the developing brain. The map visually indicates when to protect or stimulate each sense, offering clear, developmentally appropriate guidance. Printed and digital versions of the map were made accessible to families and staff, with bedside copies and a poster displayed in the unit. **Results:** A post-implementation evaluation is ongoing, but preliminary feedback suggests that the SDCM improved the family understanding of sensory developmental care. The SDCM serves as a valuable resource for promoting appropriate sensory input for preterm infants and further enhancing developmentally supportive care within the NICU.

## 1. Introduction

Infants born premature or with perinatal complications requiring intensive care are at a high risk of acquiring morbidities during their neonatal course, with varying short- and long-term health and developmental effects. Initiatives aiming to prevent major morbidities, such as severe intraventricular hemorrhage, necrotizing enterocolitis, and chronic lung disease, have been a cornerstone of quality improvement work in neonatal intensive care units (NICUs) [1], and a mantra for the core values of the NICU culture in the modern era across the world.

**Figure 1:**
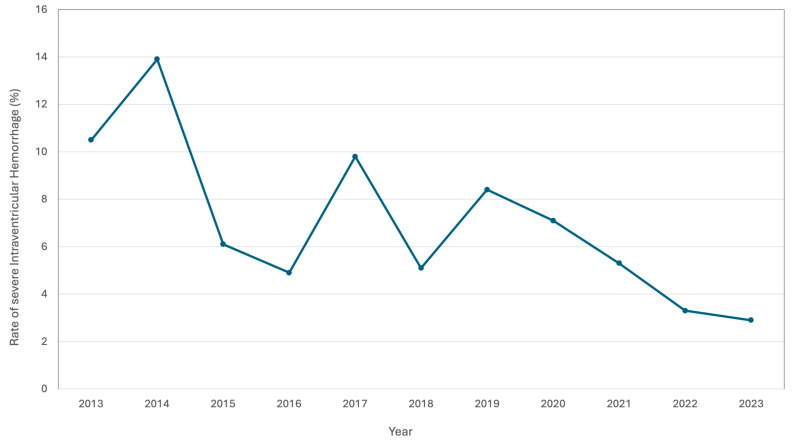
Rate of severe intraventricular hemorrhage in preterm infants <29 weeks of gestation since the implementation of the brain care bundle at Sunnybrook Health Sciences Centre in 2013 (data from the Vermont Oxford Network).

The implementation of best practices and care bundles aimed to protect infants from major morbidities have led to reduced incidents and severity of these complications [2,3]. Having successfully implemented and sustained a unit-specific brain care bundle aimed at protecting the perinatal brain from injury, referred to as neuroprotection, our quality improvement team has demonstrated a statistically significant reduction in the incidence of severe intraventricular hemorrhage in inborn preterm infants <29 weeks of gestation (Figure 1). However, there are many other factors contributing to the neurodevelopmental outcome for preterm infants [4]. Emerging literature evidence highlights the importance of neurosensory development in preterm infants and how nurturing and noxious sensory inputs can differentially affect its trajectory. Sensory development normally occurs in a developmentally timed sequence within the protective uterine environment. The various senses provide vital input about the uterine environment to the developing brain. This incoming information is vital to optimal brain development and growth, as well as attachment to the maternal caregiver. For the preterm infant, this protective environment is disrupted, and the noxious sensory input from the NICU can be detrimental to the developing brain. Positive sensory input has been shown to have beneficial effects for preterm infants and the emotional bond with their caregivers. [5]. With this concept of promoting neurodevelopment (neuropromotion) in mind, our team has shifted focus toward providing infants with positive neurosensory stimuli, especially from their parents, while maintaining neuroprotection. Termed Nurturing Encounters (NEs), positive neurosensory inputs offer balance against negative stimuli, such as pain and stress, and have led to the implementation of several changes in practice. Neuropromotion, the goal of fostering optimal neurodevelopment, has led to the development of a quality improvement program. This program aims to enhance developmental outcomes by increasing developmentally supportive interactions, supporting families during care transitions, and minimizing trauma for both families and infants.

Involving families in their children’s care in the NICU has been shown to improve the families’ experience and neonatal outcomes [6]. Family-integrated care, which has long been embraced as a model of care in our NICU, encourages families to participate in the care of their infants, engage in discussions on medical rounds, and become an integral part of the NICU team [7]. The medical team routinely provides families with information on Kangaroo Care [8,9], talking (language exposure) [10,11,12], hand hugging (nurturing touch) [13], and the association of these NE strategies to promote positive brain development. However, in a voluntary, anonymous survey, families (*n* = 10) indicated that information that they have received from health providers in the NICU was inconsistent at best and contradictory at worst. This inconsistency of information has been shown to increase the families’ anxiety, which in turn causes confusion and increased stress [14]. A review of staff education and a survey of staff (*n* = 56) knowledge demonstrated that, although staff received specialized infant development educational sessions, information on the specific stages of sensory development was lacking. Often, families and staff alike seek to gather their information from various sources, which may not be reliable and may not be in line with the opinions and evidence guiding the practices in our unit. It became clear that to promote NE and other sensory developmental activities appropriately, an educational tool for families and staff with consistent, evidence-based information utilizing shared language to protect and promote brain development across the spectrum of gestational age (GA) needed to be developed. Our innovation challenge is to utilize local expertise and resources to develop an evidence-based education tool to support family and NICU staff in providing GA appropriate, developmentally supportive encounters with the aim of improving neurodevelopmental outcomes.

## 2. Materials and Methods

This QI initiative was conducted at Sunnybrook Health Sciences Centre Neonatal Intensive Care Unit (NICU), a 48-bed, single patient room, regional tertiary care academic perinatal center located in Toronto, Ontario, Canada. Our NICU is divided into 5 patient care areas—4 pods and a resuscitation room. We care for an average of 700 infants, including micropremature infants, yearly, of whom 80% are inborn.

### 2.1. Building the Sensory Development Care Map (SDCM)

The development of the SDCM is part of a larger quality improvement (QI) project that aims to achieve optimal brain development in premature infants requiring NICU care. This project includes change ideas to sustain and improve upon practices that will prevent injury, protect the normal maturation and development of the brain, and promote positive experiences and relationships. The SDCM is one change idea created to provide accessible educational tools for staff and families to support the goal to increase the average number of NE minutes per month from a baseline of 100,000 minutes to 125,000 minutes by December 2024.

Using standardized QI methodology by employing the Plan-Do-Study-Act (PDSA) cycles of change, an interprofessional team, including a registered nurse, physiotherapist, nurse practitioner, parent partner, and neonatologist, was assembled to create a tool that is freely accessible to families, as well as staff, and fit within our unit culture, practices, and language. The team began by conducting a literature search to review the evidence and expert consensus to determine when and how senses develop across the spectrum of gestational ages. A search of MEDLINE, including Epubs ahead of print, in-process, and other non-indexed citations (1946 to 1 September 2023), Embase (1974 to 1 September 2023), and the Cochrane Central Register of Controlled Trials (1 September 2023), was performed. Search terms included sensory development, touch, tactile, vestibular, movement, olfactory, gustatory, auditory, and visual. Additionally, terms involving care and the timing of implementation within the NICU were used. For any articles that were retrieved, those reference lists were reviewed, and further articles were found. The timing of the implementation of various caregiving practices and guidelines within our own unit was also evaluated. To build on knowledge of the ontogeny of sensory development, actions to promote and protect the senses as they develop were added to the map, highlighting when it is most important to protect the senses, and when to begin offering positive stimuli that will help promote the growth and development of the senses. This evidence was cross-referenced with the common practices and language in our unit and adjusted to ensure acceptance among the team. A graphic designer was employed to design the map using bars of color gradients, with darker shades indicating the time when it is more important to protect a sense and when an action should be performed more frequently to promote the development of the sense. Another theme to highlight is that sensory development is multimodal, meaning that each sense requires the other senses to grow and develop optimally [15].

**Figure 2:**
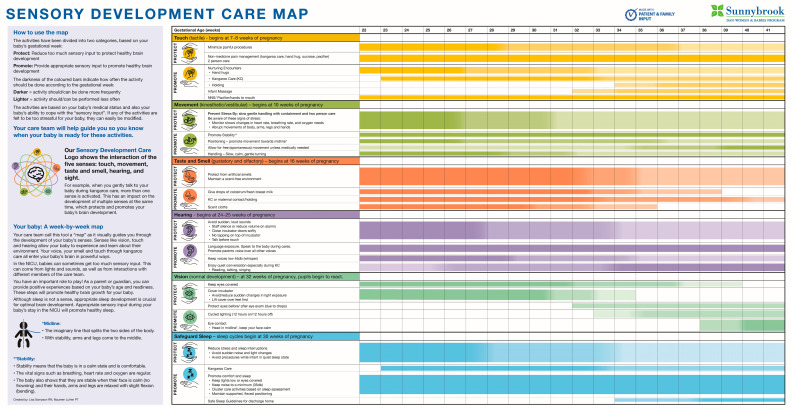
The final version of the Sensory Development Care Map (SDCM). Parents and staff are provided with a QR code link to a copy of the SDCM in portable document format (PDF). The document is housed in the Sunnybrook NICU public webpage (https://sunnybrook.ca/uploads/1/patients/mysunnybrook/sensorycaremap-acc.pdf (accessed on 31 January 2025)).

The first iteration (PDSA 1) of the tool was shown to unit staff and our family partner for input, and changes were made to comply with the culture and context of our unit. This collaboration led to the creation of a visual guide on the longitudinal sensory development for preterm infants over the spectrum of GA. Originally titled the Developmental Sensory Care Map, this guide offers practical strategies for caregivers. It empowers them to foster sensory development through appropriate stimulation while simultaneously ensuring neuroprotective care. The second iteration (PDSA 2) represented the change suggestions made by staff and our Parent Partner, including the name change to Sensory Development Care Map (SDCM). PDSA 3 involved placement of the map in 3 patient rooms along with a survey for those families in the unit to evaluate the usefulness of the tool, as well as where they would like to source this tool. At the suggestion of families and staff, a copy of the SDCM was placed at each bedside, along with a QR code link to a digital version that can be saved on personal devices to increase accessibility (Figure 2). Placement of the SDCM at each bedside allows caregivers to access appropriate information while providing care and NE so they can easily implement developmentally appropriate sensory care strategies. By providing suitable and user-friendly information to parents and caregivers, the ability to modify and provide age-appropriate sensory input for the infant becomes a strategy to reduce stress and promote healthy brain development. With this knowledge, parents can feel reassured that they are providing the best possible developmental care at each stage of their infant’s journey within the NICU. Ultimately, this level of reassurance would help to improve parental capacity, decreasing anxiety and strengthening the attachment process.

### 2.2. Measurements

To track the progress of our neuropromotion initiatives, we continuously audit the number of NEs (in minutes) being provided to each infant in the unit. Data for individual patients and aggregate data for the unit are collected, and the data and trend are collated in a quarterly communication to all unit staff so that all team members can obtain prospective feedback on the effectiveness of the entire program. To measure the success of our education initiatives, including the SDCM, and ad hoc or scheduled education for staff and families about the understanding of neuropromotion and the impact of a balanced approach to neuroprotection and developmental care, a survey of staff and families was conducted in April, 2023, to gauge the knowledge gap in this area before the implementation of the SDCM. For an evaluation of the intervention effectiveness and ongoing implementation, we used process control charts. Changes in outcomes were significant if they satisfied SPC rules (IHI rules, *p* < 0.05). For a before–after confirmation, we used a two tailed *t*-test with a *p* value of <0.05.

## 3. Results

### 3.1. Results of the NE Audits Prior to Implementation of the SDCM

**Figure 3:**
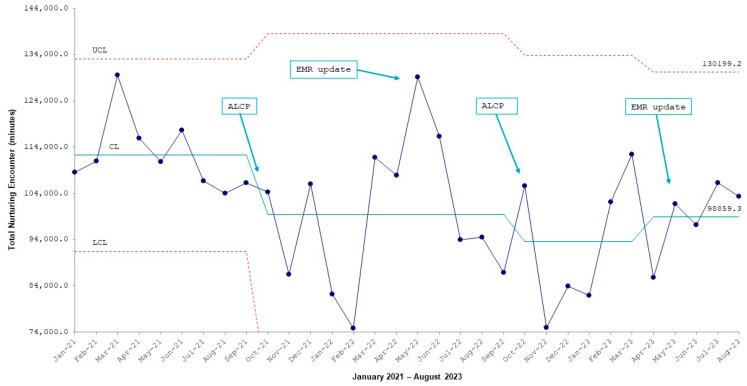
Annotated X-chart showing total nurturing encounter (NE) minutes from January 2021 to August 2023. ALCP, Autumn Leaves Cuddles Please; CL, central line; EMR, electronic medical records. LCL, lower control limit; UCL, upper control limit; Overall process is not showing change in total minutes mean.

In the first two years since the launch of the neuropromotion program, the quality improvement team implemented ongoing education sessions to staff, made improvements to the electronic medical records (EMRs) to facilitate the consistent documentation of NEs, and held special awareness-raising events called “*Autumn Leaves Cuddles Please*” as an attempt to boost the total time of NEs. However, in a review of the trend over time and according to the SPC chart, it appears that the total amount of NE time remains stagnant (Figure 3). These periodic audits have shown us, throughout the program implementation processes, that more changes are required and prompted the development of the SDCM as an educational tool.

### 3.2. Results of the Nursing Staff Survey on NEs

**Figure 4:**
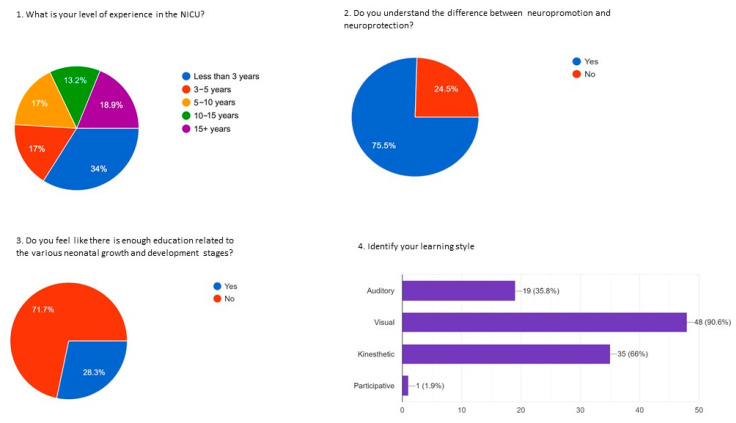
Results of the staff survey on nurturing encounters.

With the high rate of staff turnover over the past few years and the associated shift in the experience level of the nursing team, we hypothesized that more education may be required to make a significant impact on neuroprotection, neuropromotion, and developmental care. In our planned survey, amongst our nursing staff (56 respondents), 34% had less than 3 years, 17% had between 3 to 5 years, and 32.1% have greater than 10 years of experience in the NICU; 71.7% of the respondents felt that there is inadequate education and instructions on the various neonatal growth and neurodevelopmental stages. While 24.5% said they did not understand the difference between neuropromotion and neuroprotection, 45.3% felt competent to implement neuroprotective strategies based on GA, and 47.2% felt competent to implement neuropromotive strategies based on GA; 20.8% identified feeling incompetent in implementing neuroprotective or neuropromotive practices. When asked to rate their comfort level in teaching parents about infant brain development, 39.6% indicated that they feel comfortable or very comfortable, 24.5% felt moderately comfortable, and 35.8% felt uncomfortable. When asked about their learning style, staff identified that they learned primarily through visual (90.6%) and kinesthetic (66%) teaching strategies (Figure 4).

### 3.3. Results of the Parent Survey on NE

Ten NICU parents with infants born between 23–29 weeks of GA completed the survey. One identified that English is not their primary language. All respondents identified their highest level of education being College or University; 60% indicated that they had spoken to NICU team members about how to support the development of infant senses. Of these, 90% said they spoke to a nursing staff for this information. When asked about which NE terms they had heard about in the NICU, only one parent said they had heard of the term neuropromotion; two said that they had heard of neuroprotection, but 100% said they had heard of actions, such as hand hugs and Kangaroo Care. When asked how else our staff can better support parents to support their infants’ sensory development, responses clearly indicated that families are also looking for GA-appropriate information on sensory development and guidance on activities that will promote sensory development (Table 1):

### 3.4. Results of the SDCM Pre-Implementation Survey

An informal in-person survey was conducted by recruiting NICU parents, the primary end-users, to review the first iteration of the SDCM. After viewing the SDCM, parents were asked to rate how helpful the information in this tool would be for them. Using a Likert scale ranging from 0 (not helpful) to 5 (very helpful), amongst 10 parents surveyed, 6 (60%) rated the tool a 5, and 3 (30%) rated it a 4. One parent rated the tool a 3, but gave an incongruent comment that said “Personally, I think the tool explains everything more clearly for me to understand the importance of baby senses.” To maximize the accessibility of this tool for families and following their feedback, a large-sized (69”x36”) poster of the SDCM was placed in a parent area, which allowed the map to be easily accessible. In addition, copies of smaller versions of the SDCM were printed with attention to the legibility of the information for use at the infant’s bedside, and a QR code was placed at the top of all versions of the map with a link to a digital file (in PDF format) for remote use on a personal digital device.

### 3.5. Results of the SDCM Post-Implementation Survey with Staff and Families (Ongoing)

Post implementation surveys are currently underway, but preliminary responses from families (*n* = 9) have been positive (Table 2).

The survey for staff has not yet been launched, but many have verbalized appreciation for the map, in that it positively facilitates teaching families about infant neurodevelopment.

**Figure 5:**
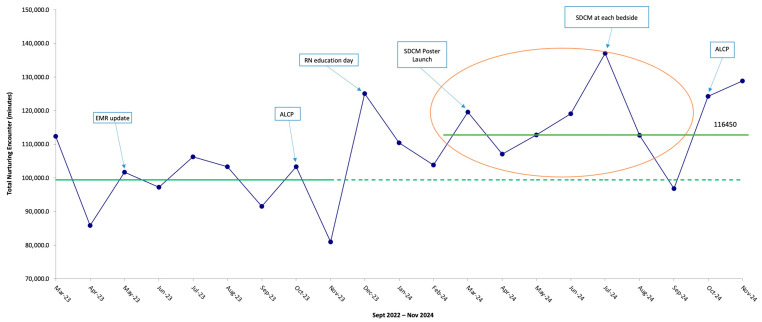
Annotated Run chart of total nurturing encounter minutes from March 2023 to November 2024, demonstrating an improvement since the launch of the Sensory Development Care Map (*p* < 0.001). EMR, electronic medical records; ALCP, Autumn Leaves Cuddles Please; RN, registered nurse.

The auditing of NE minutes has also shown a positive increase since the launch of the SDCM. (Figure 5). The pre- vs. post-implementation means of the NE minutes were 95,500 vs. 113,700 (95% CI 5875 vs. 7897, *p* < 0.001), accordingly.

## 4. Discussion

Numerous studies have addressed the impact of the NICU environment on the brain development of preterm infants. The toxic stress from painful and stressful procedures [16], in addition to the actual environment to which the infant is subjected [17], has been shown to be a contributing factor to changes in brain development of the immature preterm brain [18]. This phenomenon has been called encephalopathy of prematurity, which is manifested by diminished regional brain volumes and impaired connectivity, resulting in brain dysmaturation [19].

The neonatal intensive care mindset focuses heavily on the acute care of sick newborns, with the view that invasive interventions and diagnostic tests and procedures are lifesaving measures that are unavoidable and essential to the survival of these vulnerable patients. Changing a unit’s culture to emphasize neuropromotion is a considerable challenge and requires an awareness that noxious stimuli from pain and discomfort of medical interventions and the harsh environment of an intensive care unit are detrimental to the developing brain of preterm infants [20]. During the first two years of the neuropromotion quality-improvement program, small tests of changes were implemented. First, a unit-based practice guideline for neuropromotion was implemented with clear definitions of what constitutes an NE and to emphasize the importance of providing NEs by staff during daily care and to support families in providing NEs to their infants. An annual event to raise awareness and highlight the importance of NEs using a marathon-type celebration in the fall season called “*Autumn Leaves and Cuddles Please*” was held in the unit. Staff receive ongoing education to equip them to regularly discuss the benefits of [NE] with parents. This education emphasizes the crucial role parents play in providing these positive interactions. To objectively measure the effectiveness of our quality-improvement program, substantive customization to the electronic medical records (EMRs) was made to facilitate the documentation and audit of NEs in each patient.

As shown in Figure 3, however, the increase in the number of NE minutes was sustained, but no further improvement was demonstrated. While it is clear from the pre-implementation surveys that more education and guidance for staff and family are needed, other potential barriers to maintaining a neuropromotive culture must also be considered. Rapid staff turnover and a corresponding decrease in the level of clinical experience, an increase in acuity of illness as the proportion of extreme-low-gestational-age infants became higher, and the lack of staff resources to support families in performing NEs are confounding factors hindering the success of the neuropromotion goals, and these systematic factors must be addressed in order effect improvements in the long run.

During the implementation process of the SDCM, we have identified several challenges and limitations, including physical barriers, the inability to assess usage of the map, and human factors, such as a change in fatigue and burnout. Based on the layout of our single-family rooms, the only place that could be used for the consistent placement of the SDCM magnetic poster was on the equipment carts at the bedside. These carts are on wheels and can be moved around the room as needed. Inadvertently, this has led to the SDCM being hidden at times, especially if staff or parents had forgotten to move the poster to the other side of the cart to maintain its visibility. At present, there are no means to track how often the map is used. Team members can document in narrative form if the map has been used during education with families; however this is difficult to audit. In the future, further improvements to allow documentation of the SDCM usage in the EMR will allow us to better quantify the acceptance of the SDCM.

Lastly, rapid staff turnover, along with frequent change in equipment as a result of back orders and manufacturing delays for devices used in the NICU, have led to changes in fatigue and burnout in our unit. These frequent changes in care activities and routines have negatively impacted the attitudes of embracing new QI initiatives.

A major component of Developmentally Supportive care is the involvement of the families and providing them with the skills and knowledge to be able to interact with their infants in a developmentally and age-appropriate manner. In examining how best to do this, we reviewed the coaching literature that is being utilized in the early intervention field. Coaching in this context has been defined as “professional guidance aiming to empower caregivers to make their own decisions during daily care activities” [21]. This reinforces the principle of the integration of families as active participants and members of the NICU care team. The SDCM provides the appropriate information and knowledge and invites the collaboration of families and staff in the care of the infants.

With the visual guidance of the SDCM, families, and staff have been given an intuitive and easy-to-follow tool to follow the basic principles of coaching strategies. These principles involve goal setting, intervention planning, implementation, and evaluation [22]. The SDCM allows the families to set appropriate goals of care for their infant for a particular gestational age with age-appropriate strategies and suggestions for implementation. As the SDCM is available to families and staff alike, the bedside staff are available to provide the ongoing evaluation, guidance, and medical support during any of these activities.

## 5. Conclusions

The SDCM is an evidence-based activity guide to promote sensory development in preterm infants. This visual map is a helpful tool in the optimal brain development program to ensure that an NE is performed frequently and in a gestational-age-appropriate manner. The potential impact of this tool in improving long-term neurodevelopmental outcomes remains to be evaluated, but this project has demonstrated the collaboration among parents, staff, and developmental experts in the creation of an innovative educational tool that is accessible and an invaluable adjunct in promoting NE in our NICU.

## Figures and Tables

**Table 1:** Comments from NICU parents surveyed.

Comment
“[We would like] information about the development all of these senses and what is the appropriate time (weeks) to get started”
“How can we start developing these senses and the activities needed to be done (as parents)?”
“How can we track progress on these developments?”
“[We would like] information on how to help our baby’s senses and what not to do”
“Provide information on weekly milestones; impact of [intensive care] on development.”
“Nurses inform us and guide us”

**Table 2:** SDCM post-implementation survey—comments from NICU parents so far.

Comment
“I refer to it all the time. I don't want a ton of written material.”
“I like the map in the hallway too because I can focus on it away from the bedside.”
“I Would like someone to speak to me about what I am reading”
“It’s very helpful”
“I find it confusing with all the colours”
“If someone had told me about it right away, that would have been good.”
“First family meeting would be a good time to discuss the map.”
“The first week is overwhelming, so after that is better to go through the map.”

## Data Availability

Data presented in this article are available upon request from the corresponding author. The data are otherwise not publicly available.

## Abbreviations

The following abbreviations are used in this manuscript:

NICU	Neonatal Intensive Care Unit
NE	Nurturing Encounters
GA	Gestational Age
SDCM	Sensory Development Care Map
PDSA	Plan-Do-Study-Act
PDF	Portable Document Format
EMR	electronic medical records
QR	quick response
